# Comparison of delayed bleeding to immediate bleeding following thyroidectomy

**DOI:** 10.1038/s41598-023-44323-z

**Published:** 2023-10-26

**Authors:** Jun Sung Lee, Jin Seok Lee, Hyeok Jun Yun, Hojin Chang, Seok-Mo Kim, Yong Sang Lee, Hang-Seok Chang

**Affiliations:** Department of Surgery, Thyroid Cancer Center, Institute of Refractory Thyroid Cancer, Gangnam Severance Hospital, Yonsei University College of Medicine, 211 Eonjuro, Gangnamgu, 06273 Seoul, South Korea

**Keywords:** Surgical oncology, Thyroid cancer

## Abstract

It is important to identify risk factors for post-thyroidectomy bleeding requiring airway intervention or reoperation. Therefore, we aimed to compare the characteristics of patients with postoperative bleeding after thyroid surgery according to the period until reoperation. We conducted a retrospective study analyzing data between April 2009 and July 2022 and included 126 patients who had postoperative bleeding. The patients were grouped according to the period between thyroidectomy and reoperation due to bleeding (0 day, 1–7 days, > 7 days). We performed among-group comparisons of patient characteristics and surgical aspects, including the extent of surgery. The ratios of male–female and lateral neck dissection were higher in the post-operative bleeding group than in the group without bleeding. In the analysis of patients with postoperative bleeding, grouped according to period between thyroidectomy and reoperation, there was a significant among-group difference in the male–female ratio. The male sex was positively correlated with the reoperation period. Further, the reoperation period was also positively correlated with total thyroidectomy and lateral neck dissection and the operation time showed a significant among-group difference. Our results indicate that the male sex and lateral neck dissection are risk factors for postoperative bleeding after thyroidectomy. Furthermore, male sex, total thyroidectomy, and lateral neck dissection are risk factors for delayed bleeding. Therefore, clinicians should consider these factors for interventions against immediate or delayed bleeding after thyroidectomy.

## Introduction

There has been a steady increase in the total number of patients undergoing thyroid surgeries annually^1^. In the thyroid cancer center of Gangnam Severance hospital, 1405 patients underwent thyroid cancer surgery in 2009 and this number increased to 2861 in 2021. Accordingly, there has been an increasing interest in complications after thyroid surgery. Postoperative complications of thyroidectomy include hemorrhage, respiratory obstruction, recurrent laryngeal nerve (RLN) injury, hypocalcemia, and hypothyroidism^2,3^. However, complications after thyroid surgery are rare, especially when the procedures are performed by an experienced surgeon^4^. Nonetheless, even with the low incidence of complications, they should not be ignored, given their significance. For example, bilateral vocal paralysis due to bilateral RLN injury can cause severe consequences such as respiratory arrest, if ignored^5^. Hematoma is among the most severe complications after thyroidectomy^6^ as it can exert pressure on the trachea, the edema of trachea, and finally cause respiratory difficulties^7^. Therefore, in case of complaints with postoperative dyspnea with swelling of the surgical site, it is essential to promptly remove the hematoma. Persistent trachea pressure may lead to fatal consequences, including respiratory cardiac arrest^8^.

The risk factors for bleeding after thyroid surgery remain unclear. Several studies have reported no particular risk factors for bleeding after thyroid surgery^9^; contrastingly, other studies have proposed hypertension, wide extent of operation, Grave’s disorder, and other parameters as risk factors^10–12^. However, there have been no studies comparing the postoperative bleeding duration. Therefore, we aimed to compare patients with bleeding after thyroidectomy to those without bleeding and analyze the period until reoperation in patients with bleeding.

## Methods

**Figure 1:**
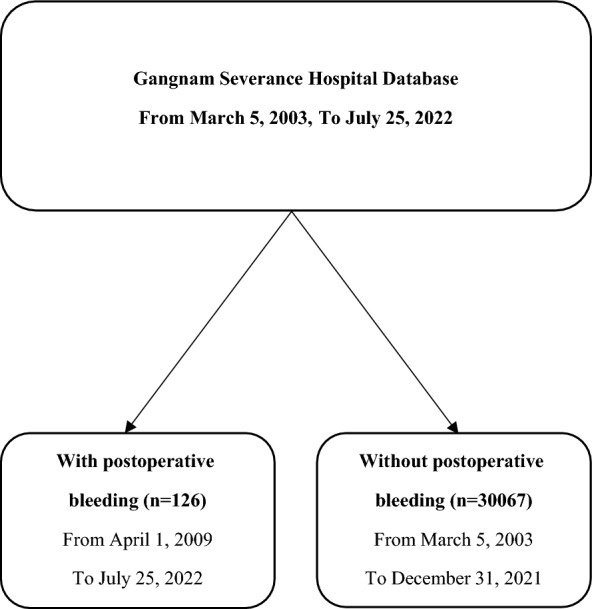
Flow diagram of patients.

**Figure 2:**
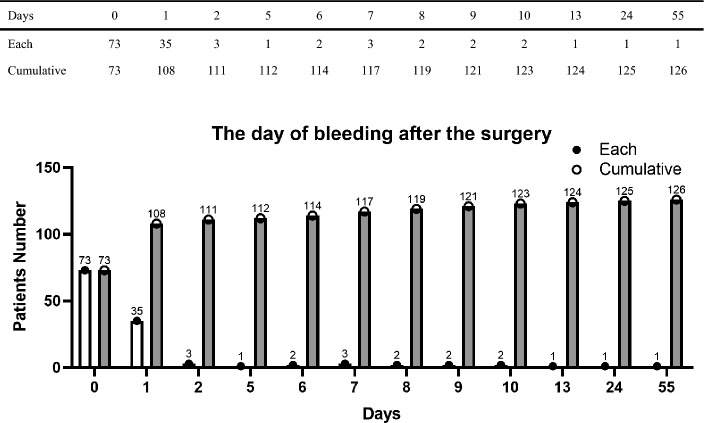
Each and cumulative patients number according to the day of bleeding after the surgery. x ratio: the day of the bleeding after the thyroidectomy, y ratio: the number of patients.

We enrolled 126 patients who underwent reoperation due to postoperative bleeding after thyroidectomy between April 1, 2009, and July 25, 2022. The patients were divided into three groups based on the duration between thyroidectomy and reoperation due to bleeding. The groups were patients who underwent reoperation due to bleeding (1) on the day of thyroidectomy (n = 73), (2) 1–7 days after thyroidectomy (n = 44), and (3) > 7 days after thyroidectomy (n = 9). In addition, we analyzed 30,067 patients without postoperative bleeding, who underwent a thyroidectomy between March 5, 2003 and December 31, 2021. The flow diagram of patients is presented in Fig. 1, and the detail about the day of bleeding after the surgery is described in Fig. 2. We collected patient clinical and surgical characteristics and analyzed them for each group.

Data on demographics, the extent of surgery, operation time, and other clinical features were also analyzed. All the included patients underwent either total thyroidectomy, less-than-total thyroidectomy, or completion of total thyroidectomy (recurred cases) with central compartment node dissection. Lateral neck node dissection was performed for cases with lateral neck node metastasis confirmed by fine-needle aspiration or intra-operative lymph node frozen pathology. Thyroiditis or extra-thyroidal cancer lesions were examined on the final pathologic reports. All statistical analyses were performed using SPSS statistical software. Graphpad Prism 8 was used for drawing Figures. Fisher’s exact or chi-square tests were used to compare categorical variables. Student’s t-test was used to compare continuous variables, which were presented as mean ± standard deviation. Statistical significance was set at P < 0.05.

This study was approved by the Institutional Review Board (IRB) of Gangnam Severance Hospital, Yonsei University College of Medicine (IRB protocol: 3-2022-1009). The study protocol was conducted in accordance with the principles of the Declaration of Helsinki. Given the retrospective nature of the study, the requirement for patient approval or informed consent was waived off by the IRB.

### Ethics approval and consent to participate

This study was approved by the Institutional Review Board (IRB) of Gangnam Severance Hospital, Yonsei University College of Medicine (IRB protocol: 3-2022-1009).

## Results

Table 1 presents the data of patients demographic and clinical characteristics. Of the 126 patients, 54 were male and 76 underwent total thyroidectomy, with nine patients showing recurrent thyroid cancer. Lateral neck dissection was performed in 41 patients. There were 35 patients with thyroiditis and 56 with an extra-thyroidal lesion of thyroid cancer. The average duration between the initial operation and reoperation due to bleeding was 1.79 days and the average operation time was 102.20 min.

**Table 1:** Baseline characteristics of the enrolled patients.

Parameter	Value(s)
Total number	126
Sex ratio (M: F)	54 (43%): 72 (57%)
Surgical extent	
Less than total	41 (33%)
Total	76 (60%)
Recurred	9 (7%)
LND	
Yes	41 (33%)
No	85 (67%)
Thyroiditis	
Yes	35 (27%)
No	84 (67%)
Unknown	7 (6%)
Extra-thyroidal lesion	
Yes	56 (44%)
No	63 (50%)
Unknown	7 (6%)
Age	50.48 ± 13.554
Height	165.74 ± 9.304
Weight	66.28 ± 12.571
BMI	23.99 ± 3.197
ASA	1.61 ± 0.604
Reoperation period	1.79 ± 5.761
Operation time	102.20 ± 64.377
Anti-coagulant or anti-platelet	
Yes	3 (2%)
No	123 (98%)
Graves	
Yes	2 (2%)
No	124 (97%)
DM	
Yes	7 (6%)
No	119 (93%)

The data are presented as n (%) or mean ± standard deviation.

Table 2 shows the comparison of patients with post-operative bleeding to those without bleeding. There was a statistical difference in the male–female ratio and number of lateral neck dissections. The number of total thyroidectomies in the post-operative bleeding group (60%) was higher than in patients without bleeding (52%) but was not statistically significant.

**Table 2:** Comparison of the ratio of male–female sex, total thyroidectomy, and lateral neck dissection between enrolled patients with and without post-operative bleeding.

Parameter	Patients without bleeding	Patients with bleeding	P value
Total number	30,067	126	
Sex			
Male	6426 (21%)	54 (43%)	** < 0.001**
Female	23,641 (79%)	72 (57%)	
Surgical extent			
Total	15,551 (52%)	76 (60%)	0.163
Others	14,516 (48%)	50 (40%)	
LND			
Yes	3231 (11%)	41 (33%)	**0.007**
No	26,836 (89%)	85 (67%)	

The data are presented as n (%).

Significant P-values (P < 0.05) are shown in bold text.

**Figure 3:**
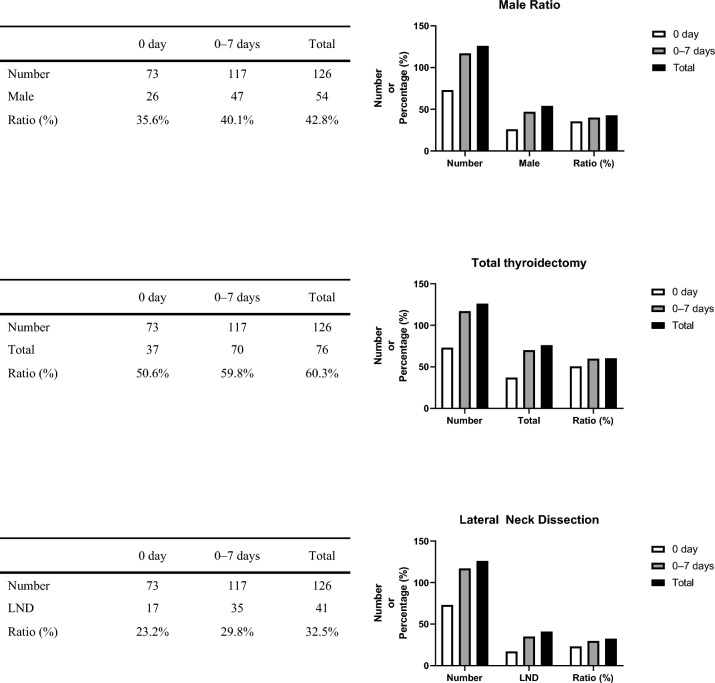
Cumulative analysis of the proportion of male, total thyroidectomy, and lateral neck dissection according to the reoperation period. x ratio: the day of the bleeding after the thyroidectomy, y ratio: the number of patients & the percentage of parameters (male, total thyroidectomy, lateral neck dissection). *Total* total thyroidectomy, *LND* lateral neck dissection.

Table 3 shows the among-group comparisons of the patients’ baseline characteristics. There was no significant among-group difference in age, body mass index, and American Society of Anesthesiologists-physical status. However, there was a significant difference in the among-group male–female ratio. The number of males was positively correlated with the reoperation period. The proportion of males in group 1 was 35% (n = 26), which was higher in groups 2 and 3 (47% and 77%, respectively). Figure 3 shows the cumulative analysis results of the male sex and percentage.

**Table 3:** Among-group comparison of the patients’ characteristics.

	Group 1 (N = 73) Mean ± SD or n (%)	Group 2 (N = 44) Mean ± SD or n (%)	Group 3 (N = 9) Mean ± SD or n (%)	P value
Age	51.86 ± 14.343	48.61 ± 12.359	45.78 ± 13.179	0.272
BMI	24.09 ± 3.469	23.85 ± 2.721	23.91 ± 3.381	0.924
ASA	1.61 ± 0.592	1.55 ± 0585	1.88 ± 0.781	0.311
Sex				
Male	26 (35%)	21 (47%)	7 (77%)	**0.039**
Female	47 (64%)	23 (52%)	2 (22%)	

The data are presented as n (%) or mean ± standard deviation.

Significant P-values (P < 0.05) are shown in bold text.

Table 4 shows the surgical characteristics of the enrolled patients. There was a significant among-group difference in the number of total thyroidectomies and lateral neck dissections. The reoperation period was positively correlated with the number of total thyroidectomies and lateral neck dissections. Total thyroidectomy was performed in 37 (51%), 33 (75%), and 6 (66%) patients in groups 1, 2, and 3, respectively. Moreover, lateral neck dissection was performed in 17 (23%), 18 (41%), and 6 (66%) patients in groups 1, 2, and 3, respectively. The cumulative analysis of the number of total thyroidectomies and lateral neck dissections are presented in Fig. 2.

**Table 4:** Among-group comparison of the surgical aspects.

	Group 1 (N = 73) Mean ± SD or n (%)	Group 2 (N = 44) Mean ± SD or n (%)	Group 3 (N = 9) Mean ± SD or n (%)	P value
Surgical extent				
Total	37 (51%)	33 (75%)	6 (66%)	**0.031**
Others	36 (49%)	11 (25%)	3 (33%)	
LND				
Yes	17 (23%)	18 (41%)	6 (66%)	**0.010**
No	56 (76%)	26 (58%)	3 (33%)	
Operation time	90.28 ± 59.261	119.47 ± 69.996	114.44 ± 60.912	**0.049**

The data are presented as n (%) or mean ± standard deviation.

Significant P-values (P < 0.05) are shown in bold text.

There was a significant among-group difference in the operation time. The average operation time in groups 1, 2, and 3 was 90.29, 119.47, and 114.44 min, respectively.

## Discussion

Complications in thyroid surgery are infrequent, especially when the procedure is performed by an experienced surgeon^3^. Nevertheless, the potential for significant morbidity is present because of the anatomical feature of the thyroidectomy operation site. Post-operative complications of thyroidectomy include hemorrhage, respiratory obstruction, RLN injury, hypocalcemia, and hypothyroidism. The reported incidence of hematoma after thyroidectomy is in about 1% of total patients^12,13^.

The risk factors of bleeding after thyroidectomy are controversial in many studies. Some studies claim the male sex as a risk factor for post-thyroidectomy bleeding^14–17^. Our results are consistent with those of previous studies. In our study, the male sex was identified as a risk factor for postoperative bleeding after thyroidectomy and delayed post-thyroidectomy bleeding. There would be several reasons that male sex was a risk factor of bleeding. First, male is usually more muscular than female anatomically, and it would increase the risk of bleeding from muscle blood oozing after the surgery. Second, muscular anatomy makes surgery difficult, so the bleeding ligation after the thyroidectomy would be difficult in male patient rather than female. Additionally, male patient would be more active than female, and excessive movement after surgery could cause bleeding at operation site. Therefore, when treating male patients, it is essential to monitor them not only for immediate postoperative bleeding but also for delayed bleeding.

Several studies have reported that total thyroidectomy is a risk factor for postoperative bleeding^12^, which could be attributed to the relatively wide site of operation compared with that of subtotal thyroidectomy. In our study, the ratio of total thyroidectomy was higher in the post-operative bleeding group than in the non-bleeding group (60% and 52%, p = 0.163). Among enrolled patients, only a few cases of postoperative bleeding resulted from a specific reason, such as vessel bleeding. Specifically, only 21 cases were due to vessel bleeding, while the remaining were due to uncertain reasons such as muscle oozing and non-specific flap bleeding. If the extent of the operation is expanded, it can be assumed that the possibility of uncertain bleeding increases. Therefore, this result may support the hypothesis that more the extent of the operation, more the likelihood of post-thyroidectomy bleeding. There is an study support this hypothesis^18^. Additionally, our findings indicated that total thyroidectomy is a risk factor for bleeding due to extended period until reoperation. If the extent of surgery is expanded, the possibility of uncertain bleeding increases; therefore, the risk of reoperation due to bleeding after long period from initial thyroidectomy is higher.

This concept similarly applies to lateral neck dissection, which involves a large surgical incision through the medial border of the sternocleidomastoid in addition to the normal thyroidectomy operation site. Same as total thyroidectomy as a risk factor, lateral neck dissection could be a risk factor of post-operative bleeding because of extend of surgery. Accordingly, lateral neck dissection was positively correlated with the risk of bleeding, especially delayed bleeding.

There was a significant among-group difference in the operation time. The average operation time in groups 1, 2, and 3 was 90.29, 119.47, and 114.44 min, respectively. This can be explained by the type of surgeries the patients underwent. The ratio of total thyroidectomy and lateral neck dissection was higher in groups 2 and 3 than in group 1. Generally, total thyroidectomy involves a longer operation time than subtotal thyroidectomy, which is further extended in case lateral neck dissection is performed. Accordingly, the operation time was longer in patients with delayed bleeding.

In our study, one case was occurred after 55 days of initial surgery. The post-operative bleeding was occurred mostly within 24 h, but sometimes it happens after a long time^19^. In our study, 55 days case had a surgery as a anaplastic thyroid cancer, and the bleeding would be occurred because of the delay of wound healing. It was a very rare case, but we should not ignore the occurrence of unusual case^20^.

Our findings indicate that male sex and lateral neck dissection were risk factors for post-operative bleeding after thyroidectomy. The results also show that male sex, total thyroidectomy, and lateral neck dissection were risk factors for delayed bleeding. Therefore, clinicians should consider these factors for interventions against immediate or delayed bleeding after thyroidectomy.

There are several limitations in our study. First, it has a limitation as a retrospective study. Second, there is no specific description about the site of post-operative bleeding. It was difficult to collect the information about the site of bleeding because we had to rely on surgical records to get information. Since most surgeries due to bleeding were emergency surgeries, the description method of each surgical record was different and the degree of accuracy was also heterogeneous.

## Data Availability

Authors are willing to make their data, analytical methods, and study materials available to other researchers on reasonable request. The data, analytical methods, and study materials during the current study are available from the corresponding author on reasonable request.

## Abbreviation

RLN: Recurrent laryngeal nerve
